# *Acrocephalus orinus*: A Case of Mistaken Identity

**DOI:** 10.1371/journal.pone.0017716

**Published:** 2011-04-22

**Authors:** Evgeny A. Koblik, Yaroslav A. Red'kin, Margarita S. Meer, Romain Derelle, Sofia A. Golenkina, Fyodor A. Kondrashov, Vladimir Yu. Arkhipov

**Affiliations:** 1 Zoological Museum of the Moscow State University, Moscow, Russia; 2 Bioinformatics and Genomics Programme, Centre for Genomic Regulation, Barcelona, Spain; 3 Laboratory of Evolutionary Genomics, Vavilov Institute of General Genetics, Russian Academy of Science, Moscow, Russia; 4 Institute of Theoretical & Experimental Biophysics, Russian Academy of Sciences, Pushchino, Moscow Region, Russia; University of York, United Kingdom

## Abstract

Recent discovery of the Large-billed Reed Warbler (*Acrocephalus orinus*) in museums and in the wild significantly expanded our knowledge of its morphological traits and genetic variability, and revealed new data on geographical distribution of the breeding grounds, migration routes and wintering locations of this species. It is now certain that *A. orinus* is breeding in Central Asia; however, the precise area of distribution remains unclear. The difficulty in the further study of this species lies in the small number of known specimens, with only 13 currently available in museums, and in the relative uncertainty of the breeding area and habitat of this species. Following morphological and genetic analyses from Svensson, *et al*, we describe 14 new *A. orinus* specimens from collections of Zoological Museums of the former USSR from the territory of Central Asian states. All of these specimens were erroneously labeled as Blyth's Reed Warbler (*A. dumetorum*), which is thought to be a breeding species in these areas. The 14 new *A. orinus* specimens were collected during breeding season while most of the 85 *A. dumetorum* specimens from the same area were collected during the migration period. Our data indicate that the Central Asian territory previously attributed as breeding grounds of *A. dumetorum* is likely to constitute the breeding territory of *A. orinus*. This rare case of a re-description of the breeding territory of a lost species emphasizes the importance of maintenance of museum collections around the world. If the present data on the breeding grounds of *A. orinus* are confirmed with field observations and collections, the literature on the biology of *A. dumetorum* from the southern part of its range may have to be reconsidered.

## Introduction

Since the publication of reviews on the distribution of birds in the former USSR [1], [2] there have been no new descriptions of species with the main breeding ranges on this territory [3]. A possible exception is *A. orinus*, the Large-billed Reed Warbler, which has been recently found to be breeding in the southern area of Tajikistan [4], however, the more precise breeding area of this species remains undetermined. This enigmatic bird has captured the attention of several researchers after two live birds were caught during non-breeding season in Thailand [5]–[6], the first such records of this species for well over a century, and which collectively proved the existence and status of *A. orinus* as a separate and non-extinct species. The recent discovery of 11 new museum specimens of *A. orinus,* including one specimen from Kazakhstan [7]–[8], as well as the capture of live individuals in northern Afghanistan [9] and Southern Tajikistan [4], established that the main breeding range of this enigmatic species is located in the broader Central Asia that includes Afghanistan [4], [8], [9]. The 11 specimens were discovered after careful analysis of *A. dumetorum* museum collections, suggesting that *A. orinus* can easily be overlooked as this species. These two factors, the sites of the collected *A. orinus* specimens in the breeding season on the south border of the territory of the former USSR and the distribution of *A. dumetorum* in the Central Asian countries, led us to examine more closely the available collections of *A. dumetorum* from the southern extension of its breeding range.

## Results

### Identification of *A. orinus* specimens

Following previous work [4], [8], [10] we hypothesized that the Large-billed Reed Warbler breeds on the territory of the former Soviet Union, but that this species was not recognized previously because its identity was mistaken with *A. dumetorum*. Thus, starting in the summer 2009 we have undertaken a large survey of specimens labeled as *A. dumetorum* in the ornithological collections of several museums located on the territories of the former USSR. Following Svensson, *et al*. [10], several morphological measurements were used to distinguish the potential *A. orinus* from among the specimens labeled as *A. dumetorum* (see Methods). Specifically, *A. orinus* was distinguished by a longer, slightly broader and duller bill, longer and more graduated tail, with the outer tail feathers substantially shorter than the central tail feathers and finally by a longer hind claw (Table 1). In total, the original morphological identification of possible *A. orinus* specimens revealed 14 high confidence candidates and three questionable ones.

**Table 1:** Biometrics for Large-billed Reed Warbler and Blyth's Reed Warbler (in mm) (original data).

	Large-billed Reed Warbler				Blyth's Reed Warbler					
	N	M	SD ±	min	max	n	M	SD ±	min	max
Bill to skull	13	**18,9**	0,55	**18,0**	**19,6**	30	**17,1**	0,48	**16,2**	**18,0**
Bill from prox. edge nostril	9	**11,7**	0,22	**11,5**	**12,0**	30	**10,7**	0,47	**9,7**	**11,4**
Hind claw	14	**7,4**	0,42	**6,9**	**8,2**	30	**6,2**	0,27	**5,7**	**6,7**
Distance between tips central and utmost tail-feathers	11	**7,5**	1,06	**6,0**	**9,4**	30	**4,9**	1,03	**3,3**	**7,5**
Tail/wing in %	11	**91,5**	1,86	**89,1**	**94,3**	30	**84,0**	0,02	**81,0**	**88,0**
Tail	11	57,3	1,51	54,3	59,2	30	53,2	1,73	50,0	58,0
Wing	14	62,8	1,66	59,4	66,5	30	63,2	1,62	60,6	66,6

**Figure 1:**
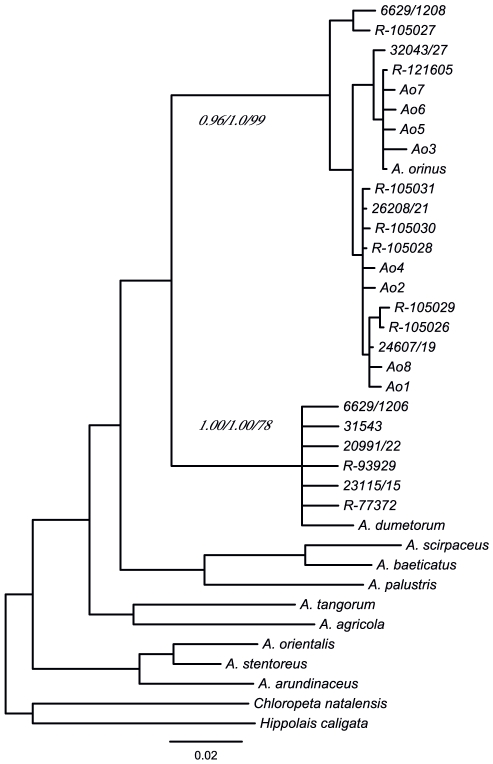
Unrooted phylogenetic reconstruction of selected *Acrocephalus* species and two outgroups. The posterior probabilities and bootstrap values are shown for Bayesian, Maximum Likelihood and Neighbor Joining methods, respectively, for *A. orinus* and *A. dumetorum* clades. The Bayesian posterior probability of all interspecies comparisons is 0.99 or greater, except the branch between *A. agricola* and *A. tangorum* (0.77). The non species name labels are museum identifiers with R 77372, R 93929, 23115/15, 20991/22 and 31543/23 corresponding to the 5 control *A. dumetorum* specimens and 6629/1206 to the low confidence *A. orinus* that the phylogenetic analysis confirms as *A. dumetorum* (Table 2).

To verify our identification we isolated genomic DNA from skin samples of the specimens identified by morphology as *A. orinus* as well as from several individuals identified as *A. dumetorum* as a control (see Methods), listed in Table 2. Out of the 17 samples of potential *A. orinus* specimens DNA isolation was successful for 12 cases, with unsuccessful isolation for one century-old sample from a high confidence *A. orinus*, two older than a century samples from low confidence specimens and two specimens from NUUz from which we did not obtain skin samples (Table 2 and 3). The isolated genomic DNA was then sequenced using primers reported by Svensson, *et al.* [8] and the obtained sequences analyzed using three different phylogenetic approaches (see Methods). The results of our phylogenetic analysis give high support for 11 out of the 12 sequenced potential *A. orinus* specimens being identified correctly. The sequences from 5 control *A. dumetorum* specimens, as well as one specimen, initially identified as a low confidence *A. orinus*, clustered with previously sequenced *A. dumetorum* (Figure 1). The tree topology of selected *Acrocephalus* species recapitulates the previously reported efforts [11], and the congruence of our tree for different methods of phylogeny reconstruction, demonstrate that the clustering of the 11 specimens with previously determined *A. orinus* sequence is highly reliable.

**Table 2:** Selected sequenced *Acrocephalus dumetorum* specimens from collections of former USSR.

Museum Number	Museum	Sex	Location	Date	Collector
R 77372	ZMMU	Male, juv.	Sory-Tash, Alay valley Kirgizstan	16.08.1935	M. Shtrom
R 93929	ZMMU	Male ad.	Batkhys Reserve, Turkmenistan	19.05.1967	L.S. Stepanyan
23115/15	ZMAU	Male, juv.	Gyazgedyk, Ashgabat Region, Turkmenistan	14.08.1948	Unknown
20991/22	ZMAU	Male ad.	Gava, Osh Region, Kirgizstan	10.08.1960	A.F. Klimenko
31543/23	ZMAU	Female ad.	Kondara, Gissar Range,Tajikistan	12.06.1978	A.M. Peklo
6629/1206	SDM	Female ad.	Kulyab (South Tajikistan)	03.05.1910	M.N. Divnogorsky

The bill morphology of the specimen that was confirmed as *A. dumetorum* from a low confidence specimen (SDM 6629/1206) was consistent with a large-billed *A. dumetorum* while the measurement of the hind claw approached lower-range measurements of *A. orinus*. In contrast, the three high confidence specimens for which we did not obtain genomic DNA sequence were morphologically more different from *A. dumetorum* (Table 1 and Table S1), suggesting that these three specimens indeed belong to the *A. orinus* and were treated as such for the purpose of further analyses.

We compared the measurements of morphological traits between the 14 *A. orinus* and 30 *A. dumetorum* specimens (Table 1). Generally, our measurements of the differences between *A. dumetorum* and *A. orinus* are broadly congruent with those reported by Svensson, *et al* [10]. However, the following quantitative differences between our dataset and the one analyzed by Svensson, *et al* [10] are apparent. The average tail length of *A. orinus* in our dataset was slightly longer (54,9±2,85 mm and 57,3±1,51 mm for Svensson, *et al* [10] and our data, respectively). However, these differences should be taken with a grain of salt as they can be caused by very small sample sizes, by substantial differences in seasonal, sex or age variation of the birds in the two samples or personal differences in measurement techniques. Otherwise, as previously noted by Svensson, *et al* [10], the two species are remarkably similar, with the upper parts of the plumage *A. orinus* being just slightly darker and the bottom perhaps slightly more yellowish.

### Geographical distribution

**Figure 2:**
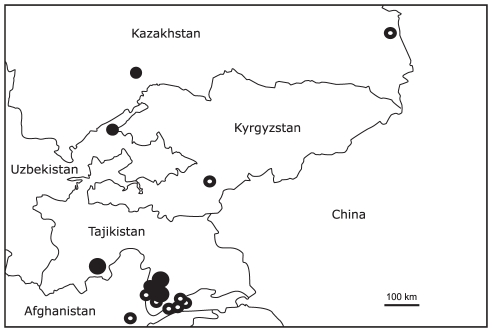
Map, showing the localities of specimens of Large-billed Reed Warbler *Acrocephalus orinus* in Central Asia (dot). The large black dots for more then two records per locality, empty dot for records known from literature.

The identification of *A. orinus* from among specimens labeled as *A. dumetorum* in several museums of the former USSR confirms the previous hypothesis that the enigmatic *A. orinus* was simply overlooked as *A. dumetorum*. Further analysis of specimens collected in the breeding season allowed us to broadly characterize the possible breeding range of *A. orinus* (Figure 2).

In the collection formerly belonged to M.A. Menzbier (now located in SDM and ZMMU) we found two adult males collected near Kulyab or Kulob (South Tajikistan) on May 19, 1910 by M.N. Divnogorsky. Curiously, on May 3, Divnogorsky collected in the same location a Blyth's Reed Warbler female, which we confirmed by genetic analysis (SDM 6629/1206).

In the collection of ZMMU we found 7 specimens of Large-billed Reed Warbler. One of them was an adult male collected by L.S. Stepanyan on May 27, 1971 near Shirvoz, in the Barvoz forest of the Shakhdara River valley, Gorno-Badakhshan (Kuhistoni-Badakhshon) in the Autonomous Province (GB AP) of Tajikistan. Also, a series of 4 adult males and 2 adult females were collected by B.N. Gurov on June 13–22 1976 near Barsem Village GB AP of Tajikistan.

Based on the catalog of ornithological collections of ZMAU [12] we studied a small series (n = 8) of *A. dumetorum* from the south of Central Asia. Among them we identified three adult males as *A. orinus*. Two of these birds were collected by V.M. Loskot, on May 31, 1968 in Shakhdara River valley near Khorog (Khorugh) GB AP of Tajikistan, and one by N.L. Klestov on June 19, 1979 in Barvoz forest, Shakhdara, GB AP of Tajikistan.

In the collection NUUz, that maintains the collection of N.A. Zarudny, we identified two adult males as *A. orinus*, both collected by D.N. Kashkarov. One was collected on May 19, 1926 near Biylyu-Kul Lake, Jambyl Province of Kazakhstan and another on June 23, 1927 near Chimgan or Chimgon in Tashkent Province of Uzbekistan.

It is possible that two young males identified as low confidence *A. orinus* from the ZMMU collections are true specimens of *A. orinus*, one (R–36373) collected on August 2, 1879 on the Kunges river (tributary of r. Ili, Eastern Tyan-Shan mountains, China) and one (R–65803) collected on August 21, by an unidentified collected prior to 1918 as judged by the style of the writing on the label. Because of underdeveloped features of these young males it is not possible to make this distinction conclusively without DNA analysis, which at this point has not been attempted due to the difficulty in extracting DNA from such old samples. Therefore, we have not included these two specimens on our analyses.

## Discussion

Based on the geography of the *A. orinus* specimens collected in May through June, which is the likely breeding season, the breeding range of this species in Central Asia spans mountainous regions, 900–3000 meters above sea level, of Tajikistan (except the Eastern Pamyr mountains), Kyrgyzstan, eastern Uzbekistan and south-eastern Kazakhstan (Figure 2). The most north-western and lowest point of collection is on the Biylyu-Kul Lake, 430 m. above sea level. The most specimens, at least 10, were collected on the western Tajik part of Gorno-Badakhshan Autonomous Province (river valley of Shakhdara, Tajikistan) that were collected between 1968 and 1979, which represents the most recent collections among our sample. In light of the recent findings of territorial males of *A. orinus* in the Afghan part of Badakhshan [9] the main part of the breeding range may be located in the watershed of Panj River.

The breeding range of *A. orinus* appears to overlap with the area in Central Asia that has always been attributed to the range of *A. dumetorum* [13]. Thus, a re-assessment of the literature on the biology of *A. dumetorum* in this region is called for, as some descriptions of *A. dumetorum* may actually be of *A. orinus*. Similarly, the issue of the zones of sympatry of these two species needs to be addressed.

**Figure 3:**
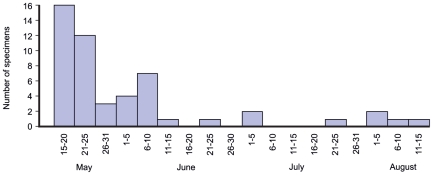
Distribution of the 53 *A.* *dumetorum* specimens collected in the likely breeding season (from May 15 to August 15) in Central Asia and South Kazakhstan. All other *A. dumetorum* specimens from this area were collected either earlier or later in the year.

The earliest registrations of *A. orinus* in the breeding season in the Central Asian region was on May 19 and more than half of specimens (8) were collected in June (Table 3). In museum collections we checked the collection dates of *A. dumetorum* specimens from the same region (n = 95) and found that the earliest registrations were in the last days of April (3 specimens), in May the largest number of specimens were collected (53), in June only 13 specimens were obtained and, finally, just three in July. Between 6 and 23 of July not a single bird was collected, and more than 20 birds were collected in August, September and November. In the mountainous part of the region (Pamir-Alay, Tyan-Shan) in June and July only 4 birds were collected. In Figure 3 we provide information on the number of *A. dumetorum* specimens collected in what is likely to be the breeding season (from May 15 to August 15) in Central Asia and Southern Kazakhstan. Thus, the *A. dumetorum* collected in the area of likely *A. orinus* breeding range are most likely migrant individuals passing through or non-breeding individuals.

**Table 3:** *Acrocephalus orinus* (n = 14), from collections of former USSR.

Museum Number	Museum	Sex	Coordinates	Location	Date	Collector
R–105026	ZMMU	Male	N 37°33′E 71°44′	Barsem, GB AP of Tajikistan	22.06.1976	B.N. Gurov
R–105027	ZMMU	Female	N 37°33′E 71°44′	Barsem, GB AP of Tajikistan	16.06.1976	B.N. Gurov
R–105029	ZMMU	Male	N 37°33′E 71°44′	Barsem, GB AP of Tajikistan	13.06.1976	B.N. Gurov
R–105031	ZMMU	Male	N 37°33′E 71°44′	Barsem, GB AP of Tajikistan	14.06.1976	B.N. Gurov
R–105030	ZMMU	Male	N 37°33′E 71°44′	Barsem, GB AP of Tajikistan	16.06.1976	B.N. Gurov
R–105028	ZMMU	Female	N 37°33′E 71°44′	Barsem, GB AP of Tajikistan	13.06.1976	B.N. Gurov
R–121605	ZMMU	Male	N 37°11′E 71°51′	Barvoz forest, Shakhdara, GB AP of Tajikistan	27.05.1971	L.S. Stepanyan
R–21169*	ZMMU	Male	N 37°55′E 69°47′	Kulyab (South Tajikistan)	19.05.1910	M.N. Divnogorsky
6629/1208	SDM	Male	N 37°55′E 69°47′	Kulyab (South Tajikistan)	19.05.1910	M.N. Divnogorsky
32043/27	ZMAU	Male	N 37°11′E 71°51′	Barvoz forest, Shakhdara, GB AP of Tajikistan	19.06.1979	N.L. Klestov
24607/19	ZMAU	Male	N 37°28′E 71°35′	Khorog (Khorugh), Shakhdara, GB AP of Tajikistan	31.05.1968	V.M. Loskot
26208/21	ZMAU	Male	N 37°28′E 71°35′	Khorog (Khorugh), Shakhdara, GB AP of Tajikistan	31.05.1968	V.M. Loskot
16980*	NUUz	Male	N 43°04′E 70°43′	Biylyu-Kul Lake, Jambyl Province, Kazakhstan	19.05.1926	D.N. Kashkarov
16976*	NUUz	Male	N 41°33′E 70°00′	Chimgan, Tashkent Province, Uzbekistan	23.06.1927	D.N. Kashkarov

*DNA analysis was not taken.

A literature review of the breeding registrations of *A. dumetorum* in mountainous regions of Central Asia revealed only a few possible registrations of this species. Shnitnikov [14] and Yanushevich, *et al.* [15] do not report any definite evidence of breeding of *A. dumetorum* in the mountains of south Kazakhstan and Kyrgyzstan. However, recent observations [16], establish this species as “common breeding species” for the Issyk-Kul valley of Kyrgyzstan. Unfortunately, no good descriptions for these records were done. Two “nestlings” were collected by N.A. Severtsov [17] on the upper parts of the Ili river, located on the territory of modern day China, however, the late data of registration (August 28) suggests that these individuals were rather well flying juveniles, which could have been migrant individuals. We believe that the author has made an error when referring to these individuals as "nestlings". In addition, one more registration of a flightless nestling was made in Almaty [17]. Ivanov [18] claimed that there were no definite data on breeding of *A. dumetorum* in the Pamir-Alai mountains. However, Meklenburtsev [19] was confident in the breeding of *A. dumetorum* in the valley of Kashdarariya and N.A. Zarudny [18] claimed to have observed breeding of this species in the valley of Kafirnigan river, near Dzhilikul and in the mountains east of Kulyab. Abdusalyamov [20] describes registrations of breeding pairs of *A. dumetorum* in the valleys of Syrdariya and Zeravshan rivers, broods and nestlings on the Shakhdara river and Darvaz mountain range, but describes only a single nest from Sary-Kumysh, Syrdariya river.

Overall, the few reliable literature descriptions of the geographical locations of breeding sites of *A. dumetorum* in Central Asia coincide with collection locations of specimens we identify as *A. orinus*. Authors frequently describe the same sites of breeding of *A. dumetorum* as the sites of collection of our confirmed *A. orinus* specimens, such as Shakhdara river valley, Barvoz forest and East of Kulyab.

Thus, it is conceivable that reports of nesting of *A. dumetorum* in the mountainous regions of Central Asia and southern Kazakhstan are reports on the nesting of *A. orinus*. However, it does not appear possible to resolve the issue of sympatry of these two species based solely on existing ornithological collections because most *A. dumetorum* specimens from this region were collected in the period of seasonal migrations and the few adult specimens collected in the breeding season may correspond to non-breeding individuals that did not fly further north to their own breeding grounds. The only registration of nesting of *A. dumetorum* south of Central Asia was made in 1875 in the range leading up to the Himalayan Mountains in India [21], when a specimen was collected nearby a nest, which is now available in the NHM collection as mentioned in [10]. Careful reading of Anderson's work leads us to conclude that this registration is a mistake because the description of the nest does not correspond to the known nest structure of *Acrocephalus* genus. Thus, the two close species *A. orinus* and *A. dumetorum* may not be sympatric species, as is the hypothesis of several authors [8], [10], but are likely to be allopatric.

The emergence of the enigma surrounding the identity and distribution of the Large-billed Reed Warbler appears to have been fueled, at least in part, but the segregation imposed by the Iron Curtain, leading to the linguistic isolation of Soviet ornithologists from western literature on one hand, and the physical isolation of Western ornithologists, which prevented them from traveling to the regions in question, on the other.

## Materials and Methods

### Specimen identification and morphological analysis

All measurements were done following the recommendations of Svensson [22]. In total, we identified 14 *A. orinus* specimens in the collections of Zoological Museum, Moscow Lomonosov State University (ZMMU), State Darwin Museum, Moscow (SDM), Zoological Museum, National Museum of Natural History of the National Academy of Sciences of Ukraine, Kiev (ZMAU) National University of Uzbekistan (NUUz) in addition to the 95 specimens of *A. dumetorum* collected from different locations in Central Asia and Southern Kazakhstan, including 33 from Turkmenistan, 19 from southern regions of Uzbekistan, 12 samples from Tajikistan, 8 from Kyrgyzstan, 20 from Southern Kazakhstan and 3 from North-Western China. All measurements were taken by Yaroslav Red'kin, with the exception of all measurements of specimens from NUUz, which were performed by R.D. Kashkarov, A.A. Atakhodjaev, I. Atamuradova, N. Azimov and Sh. Ziyovatdinov. For sequence comparison we also selected five *A. dumetorum* specimens from Central Asia and one additional *A. dumetorum* specimen that was initially identified as a low-confidence *A. orinus* (Table 2).

### DNA extraction and sequencing

The samples were obtained as a small skin fragment cut from the incision on the lower half of the keel from the bird skin of *Acrocephalus sp.* specimens. We followed the protocol for DNA extraction from using a QIAamp DNA Mini Kit, Qiagen from Rogaev, *et al.* [23] including the use of the same clean room facilities of Rogaev's lab [23] that has never been used for the isolation of *Acrocephalus* species. The extraction was performed according to DNA purification from Dried Blood Spots Qiagen protocol, provided by the manufacturer, except that the volume of all buffers used before washing steps and incubation times were increased. Briefly, samples were placed in 2-mL tubes containing 360 µL of buffer ATL and incubated at 85°C for 15 min. Then 40 µL of proteinase K was added, and after vortexing, samples were incubated at 56°C for 20 min. Then 400 µL of buffer AL was added and samples were incubated at 70°C for 15 min, mixed with 400 µL of ethanol and supernatants were applied to the spin columns. After standard washing steps with AW1 and AW2 buffers DNA was eluted in 35 µL of buffer AE, first elution, and 50 µL of water, second elution. PCR amplification was performed according to standard protocol using primers reported by Svensson, *et al*. [8]. GenBank Accession Numbers of obtained sequences are HM352773–HM352789.

### Phylogenetic analysis

The obtained sequences were aligned using muscle [24] and the multiple alignment was then used to infer phylogenetic relationships between previously determined sequences of *A. orinus* (EU490497.1) and *A. dumetorum* (FJ883028.1) as well as selected Acrocephalidae, including *A. agricola* (FJ883021.1), *A. arundinaceus* (GQ242151.1), *A. baeticatus* (FJ883024.1), *A. orientalis* (FJ883034.1), *A. palustris* (FJ883036.1), *A. scirpaceus* (FJ883039.1), *A. stentoreus* (FJ883031.1) and *A. tangorum* (FJ883041.1) using the closely related *Chloropeta natalensis* (DQ008523.1) and *Hippolais caligata* (AJ004793.1) as outgroups. In addition, we included in our phylogenetic analysis the sequences of 8 *A. orinus* specimens reported by Ayé, *et al.* [4]. Sequences specimens from Svensson, *et al.* [8] were not publicly available. For Bayesian inference we used MrBayes 3.1 [25], with 1,000,000 iterations and HKY85 model assuming a gamma-distribution of substitution rates across sites. For Maximum Likelihood we used Phyml 3.0 [26] with HKY85 model, estimated transition to transversion ratio and gamma-distribution of substitution rates across sites. For Neighbor Joining approach we used MEGA 4 software [27] with 100,000 bootstrap replicates and the Tajima-Nei model. *A. orinus* specimens that were clustered on the same branch of the reconstructed phylogeny did not correlate with the geographical location of their collecting (data not shown).

## Supporting Information

Table S1 **Biometrics for Large-billed Reed Warbler specimens (in mm) (Original data).**
(DOC)
