# Supplementary material for: Acrocephalus orinus: A Case of Mistaken Identity
Source: PLoS One. 2011 Apr 22;6(4):e17716. doi: 10.1371/journal.pone.0017716 (PMC3081296; doi:10.1371/journal.pone.0017716)
Supplement: Table S1 — Biometrics for Large-billed Reed Warbler specimens (in mm) (Original data). (DOC) [file pone.0017716.s001.doc]

| Museum Number | R-21169 | R-105026 | R-105027 | R-105029 | R-105031 | R-105030 | R-105028 | R-121605 | 6629/1208 | 32043/27 | 24607/19 | 26208/21 | 16980 | 16976 |
| --- | --- | --- | --- | --- | --- | --- | --- | --- | --- | --- | --- | --- | --- | --- |
| Sex | Male | Male | Female | Male | Male | Male | Female | Male | Male | Male | Male | Male | Male | Male |
| Wing | 61,8 | 64,0 | 62,0 | 59,4 | 62,5 | 63,8 | 60,6 | 63,4 | 63,3 | 63,0 | 62,8 | 63,7 | 66,5* | 62,7* |
| Distance from first secondary to wing tip | 12,4 | 14,0 | 13,3 | 13,0 | 13,3 | 11,8 | 12,2 | 13,3 | 12,8 | 13,3 | 13,6 | 14,7 |  |  |
| Distance from second primary to wing tip | 4,8 | 4,6 | 5,0 | 5,0 | 6,1 | 5,5 | 6,2 | 5,2 | 5,0 | 4,6 | 5,4 | 5,2 |  |  |
| Tail |  | 58,3 | 58,1 | 56,0 | 56,6 | 58,6 | 54,3 | 59,0 | 59,2 | 56,1 | 56,9 | 57,0 |  |  |
| Tail/wing in % |  | 91,1 | 93,7 | 94,3 | 90,6 | 91,9 | 89,6 | 93,1 | 93,5 | 89,1 | 90,6 | 89,5 |  |  |
| Distance between tips central and outermost tail-feathers |  | 7,5 | 9,4 | 6,3 | 7,3 | 8,7 | 6,8 | 8,8 | 7,0 | 6,6 | 6,0 | 7,6 |  |  |
| Tarsus | 23,1 | 23,2 | 22,8 | 22,1 | 23,3 | 23,2 | 23,4 | 23,5 | 24,2 | 22,8 | 22,7 | 23,0 |  |  |
| Hind claw | 8,2 | 7,5 | 7,3 | 7,0 | 7,0 | 7,3 | 6,9 | 7,4 | 8,2 | 7,0 | 7,0 | 7,3 | 7,8* | 7,5* |
| Bill to skull | 19,6 | 19,6 | 18,6 | 18,0 | 19,1 | 19,1 | 18,3 | 18,8 | 19,2 | 19,1 | 19,3 |  | 18,4* | 18,0* |
| Bill to nostril | 10,3 | 10,4 | 10,1 | 9,7 | 9,8 | 10,0 | 9,6 | 9,9 | 9,8 | 9,6 | 10,1 |  |  |  |
| Bill from prox. edge nostril | 12,0 | 12,0 |  | 11,5 | 11,6 | 11,5 |  | 11,7 | 11,8 | 11,5 | 11,8 |  |  |  |
| Bill width prox. edge nostril | 5,0 | 4,7 | 4,4 | 4,5 | 4,2 | 4,7 | 4,6 | 4,7 | 4,5 | 4,5 | 4,6 | 4,4 | 5,0* | 4,8* |
| Bill depth at feathering) | 3,1 | 3,3 | 3,4 | 3,7 | 3,4 | 3,3 | 3,3 | 3,4 | 3,1 |  | 3,6 |  |  |  |
| Weight |  |  | 12,4 | 10,1 | 11,5 | 11,8 | 11,6 |  |  |  |  |  |  |  |
